# How to help researchers in palliative care improve responsiveness to migrants and other underrepresented populations: developing and testing a self-assessment instrument

**DOI:** 10.1186/s12904-019-0470-1

**Published:** 2019-10-21

**Authors:** M. Torensma, B. D. Onwuteaka-Philipsen, K. L. Strackee, M. G. Oosterveld-Vlug, X. de Voogd, D. L. Willems, J. L. Suurmond

**Affiliations:** 1 Department of Public Health, Amsterdam UMC Expertise center for Palliative Care and Amsterdam Public Health Research Institute, Amsterdam UMC, University of Amsterdam, Meibergdreef 9, Amsterdam, Netherlands; 2 Department of General Practice, Amsterdam UMC Expertise center for Palliative Care and Amsterdam Public Health Research Institute, Amsterdam UMC, University of Amsterdam, Meibergdreef 9, Amsterdam, Netherlands; 3 Department of Public and Occupational Health, Amsterdam UMC Expertise center for Palliative Care and Amsterdam Public Health Research Institute, Amsterdam UMC, Vrije Universiteit Amsterdam, de Boelelaan, 1117 Amsterdam, Netherlands

**Keywords:** Palliative care research, Responsiveness, Self-assessment instrument, Migrant patients, Underrepresented populations

## Abstract

**Background:**

European migrant populations are aging and will increasingly be in need of palliative and end of life care. However, migrant patients are often underrepresented in palliative care research populations. This poses a number of drawbacks, such as the inability to generalize findings or check the appropriateness of care innovations amongst migrant patients. The aim of this study was to develop a self-assessment instrument to help palliative care researchers assess and find ways to improve their projects’ diversity responsiveness in light of the aging migrant population, and determine whether in addition to older migrants other groups should be included in the instrument’s focus.

**Methods:**

After developing a concept instrument based on the standards for equity in healthcare for migrants and other vulnerable groups, literature review and interviews with palliative care researchers, we conducted a Delphi study to establish the content of the self-assessment instrument and used think aloud methods in a study involving seven projects for usability testing of the self-assessment instrument.

**Results:**

A Delphi panel of 22 experts responded to a questionnaire consisting of 3 items concerning the target group and 30 items on diversity responsiveness measures. Using an a priori set consensus rate of 75% to include items in the self-assessment instrument, experts reached consensus on 25 out of 30 items on diversity responsiveness measures. Findings furthermore indicate that underserved groups in palliative care other than migrant patients should be included in the instrument’s focus. This was stressed by both the experts involved in the Delphi study and the researchers engaged in usability testing. Usability testing additionally provided insights into learnability, error-rate, satisfaction and applicability of the instrument, which were used to revise the self-assessment instrument.

**Conclusions:**

The final self-assessment instrument includes a list of 23 diversity responsiveness measures to be taken at varying stages of a project, and targets all groups at risk of being underrepresented. This instrument can be used in palliative care research to assess diversity responsiveness of projects and instigate action for improvement.

## Background

The changing age structure of migrant populations across Europe calls for consideration of the particular needs of older migrants in health service provision [1]. Older migrants experience frailty and poorer health outcomes compared to non-migrants [1, 2]. As a consequence they are expected to gradually need more healthcare, including palliative and end of life care. However, studies have shown low use of palliative care services and higher levels of dissatisfaction with these services amongst this group [3, 4].

In the Netherlands, older migrants with a non-western background currently make up 4 % of the population of adults aged 65 and older. By 2060, their number is expected to have increased to 17% [5]. These older migrants are predominantly of the first generation and have a Surinamese, Turkish or Moroccan background. Perspectives on what constitutes good palliative care have been found to differ between terminally ill patients with a Turkish or Moroccan background and their Dutch care providers [6, 7]. Patients with a Turkish or Moroccan background generally preferred not to have an infaust diagnosis or prognosis disclosed in order to keep hope alive, had a preference for maximum and curative treatment until the end of life, as well as a strong preference for family care [6, 7].

Culture, diversity, and their operationalization have previously been identified as a priority for research in palliative care in Europe in order to address questions on differing needs and appropriateness of services [8]. However, we perpetually do not see migrant patients represented in palliative care research populations. Researchers across the health related fields have reported difficulties engaging migrant patients as well as other socially disadvantaged groups, and have attributed this to barriers with regards to sampling, recruitment, gaining consent, data collection, intervention delivery and retention [9]. In palliative care research, engaging migrant patients may additionally be difficult because patients’ preference for non-disclosure and curative treatment complicate discussion of palliative care. The underrepresentation of migrant patients poses a number of drawbacks, such as the inability to generalise findings or check the appropriateness of care innovations amongst migrant patients [10].

In the Netherlands, a considerable amount of palliative care research is conducted as part of the national program for palliative care innovation (*Palliantie. Meer dan Zorg*), funded by The Netherlands Organization for Health Research and Development. Over 60 research, intervention and education projects that aim to improve palliative care have been initiated between the start of this program in 2014 and the moment of study. In order to ensure representation of migrant patients in these projects researchers ideally should ensure their projects are responsive to patient diversity.

Responsiveness refers to a concept of patient experience that includes not just the interpersonal process between practitioner and patient, but the interaction between the health system and the population it serves [11]. Several strategies to improve responsiveness to patient diversity exist for the context of health care organizations [12]. The standards for equity in healthcare for migrants and other vulnerable groups, for example, are an elaborately tested self-assessment instrument that helps organisations assess their capacity to improve accessibility and quality of care for migrants and other vulnerable groups [13].

The aim of this study was to develop a self-assessment instrument to help researchers assess and find ways to improve their palliative care projects’ responsiveness to diversity, in light of the aging migrant population. It therefore predominantly concerns migrants of the first generation, and we use the term migrant to refer to persons who changed their country of residence, irrespective of the reason for migration or legal status, but not their offspring born in the country of settlement. We used the standards for equity in healthcare for migrants and other vulnerable groups – from here on equity standards – as a basis to develop a pilot instrument. The equity standards, however, have been developed for the context of healthcare organisations. We therefore adjusted the standards to be applicable for the context of palliative care research projects. Furthermore, the equity standards focus on migrants and other vulnerable groups while our aim was to develop an instrument that focuses on migrants only. We therefore also aimed to assess whether the pilot instrument’s focus was appropriate. This lead to the following research questions:

1. What should be included in a self-assessment instrument evaluating the diversity responsiveness of palliative care projects?
2. On which groups should a self-assessment instrument evaluating the diversity responsiveness of palliative care projects focus; should other groups, in addition to migrants, be included?
3. How do users evaluate the usability of this self-assessment instrument for improving the diversity responsiveness of palliative care projects?

## Methods

We developed a pilot instrument which we tested in two ways simultaneously. We used a Delphi study to test the content of the instrument and think-aloud methods to test the usability of the instrument.

### Development of the pilot instrument

**Fig. 1:**
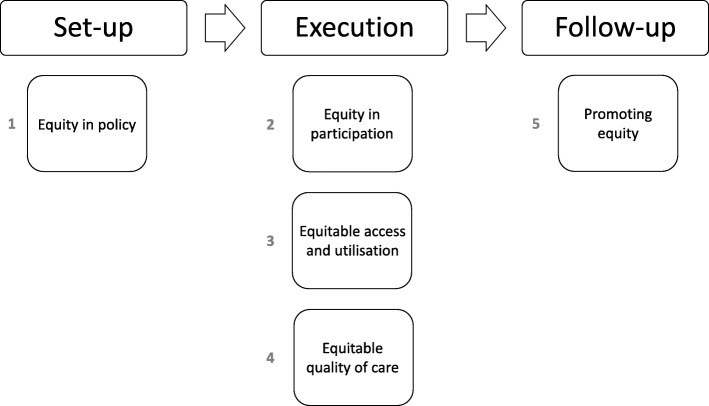
Framework for responsiveness in palliative care projects based on the domains of the equity standards

We developed the pilot instrument in two steps. First, we read academic literature on strategies to increase responsiveness to patient diversity as well as proposals of projects in the national program for palliative care innovation to establish a framework for diversity responsiveness in palliative care projects. We focused this initial framework on the domains of the equity standards: equity in policy; equitable access and utilisation; equitable quality of care; equity in participation; and, promoting equity (see Fig. 1) [13]. Secondly, we tested the applicability of this framework in semi-structured interviews with researchers involved in five palliative care projects. A description of the projects can be found at the description of the sample under section 2.2 (usability testing) of this article. During the interviews we discussed the perceived relevance and feasibility of each domain of the equity standards in relation to the projects. The insights we gained from analysis of the interview transcripts helped us develop the pilot instrument.

The pilot instrument consisted of a general introduction and instructions for use, followed by 20 diversity responsiveness measures structured in sections of the project stages: set-up; execution; and, follow-up (Table 1). Each measure could be assigned a score between one and five to describe its level of implementation in the project. Each section ended with a textbox where areas for improvement could be described. Lastly, the instrument contained a number of tips and recommendations for improvement of diversity responsiveness.

**Table 1:** Diversity responsive measures included in the pilot instrument

1.	The project set-up
1.1	Gathering insight into the diversity of the patient population
	We gather insight into the diversity of the patient population via literature or empiric research. The demographic composition of the patient population; differences in prevalence and care needs based on ethnicity and intersecting factors.
	We explicitly describe the diversity of the patient population in the project proposal. The diversity of the patient population is described; choices (not) to include patients with a non-western migration background are justified; when the choice is made to not actively include patients with a non-western migration background, implications for the outcome of the project are described in the project proposal.
1.2	Monitoring the engagement of a diverse patient population
	We include ethnicity as a variable in our research project. Patient ethnicity will be registered; subgroup analysis will be included in the data analysis plan with the aim to determine whether outcomes differ between groups based on ethnicity and intersecting factors.
	We monitor differences in care needs. With the help of existing monitor and registration systems we will evaluate the care for patients with a non-western migration background and their relatives before, during and after the project.
1.3	To establish a representative project team
	We secure knowledge on the topic of palliative care for patients with a non-western migration background within out project team. The project team knows who the relevant partners are in the field of diversity in palliative care; partners are engaged from the start; agreements are made about possible contributions.
2.	The project execution
2.1	Engaging patients with a non-western migration background
	We gather input from patients (panels or organizations) during various project stages. The patients whom we engage are representative for the patient population. E.g. when testing questionnaires or patient information materials we engage patients of varying educational levels.
	We identify and overcome barriers for participation by patients with a non-western migration background. We gather information on possible barriers amongst patient organizations representing patients with a non-western migration background.
2.2	Access to (care within) the project
	We consider which patients we reach when selecting healthcare organizations where our study will be implemented. There is geographical variety in locations where the project is implemented; when none of the locations are an entry into communities of patients with a non-western migration background, we search for additional sites.
	We work to identify and overcome gatekeeper bias to patient enrollment, for example amongst healthcare professionals. We engage healthcare professionals in the research project to increase awareness on the importance of enrolling patients with a non-western migration background; we circumvent gatekeeper bias by using patient information materials that are given to the patient directly.
	Patient information materials within and about our project are understandable. Patient information materials are basic and visual; if necessary, patient information materials are translated and adapted to increase cultural sensitivity.
	Measurement instruments used in our project are appropriate for a diverse patient population. When available, we use measurement instruments that have been tested on cultural validity; measurement instruments developed within our project are tested amongst a diverse group of patients.
	If necessary, we employ ethnically matched interviewers to conduct consent procedures, questionnaires or interviews verbally and in the language of the patient.
	With our project we contribute to increased access to palliative care for patients with a non-western migration background. Within the project we work to educate patients with a non-western migration background and their communities; within the project we offer help with patient navigation through the palliative care system.
2.3	Quality of care within the project
	We raise awareness on the increasing diversity of the patient population amongst healthcare organizations and healthcare professionals involved in the project. Healthcare organizations and healthcare professionals involved in the project are made aware of the diversity of the palliative care patient population and potential differences in understanding of what constitutes ‘good palliative care’; healthcare organizations and healthcare professionals involved in the project are encouraged to work in a culturally competent fashion.
	We offer training in cultural competencies to healthcare organizations and healthcare professionals involved in the project. Cultural competencies are included as part of training provided in the project; additional training on cultural competencies is offered; healthcare organizations and healthcare professionals involved in the project are referred to external providers of training in cultural competencies.
	Diversity in the workforce is a point of concern within our project (applicable when a project requires the formation of new care structures). In healthcare organizations with an (ethnically) diverse patient population, this diversity is mirrored in the workforce.
3	Project follow-up
3.1	Implementation of project results
	We work to sustain awareness on and efforts for diversity of the patient population in the palliative care innovation resulting from our project. During the project we think about ways to sustain the palliative care innovation, including efforts to increase its responsiveness to patients with a non-western migration background, after the project has ended.
	We advise responsiveness to patients with a non-western migration background in our recommendations or the implementation of the project in new locations.
	We share relevant findings about patients with a non-western migration background as results from the project. Where appropriate we report findings on differing outcomes between patient groups based on ethnicity and intersecting factors; We consult patient organizations representing patients with a non-western migration background on appropriate ways to share these results.
3.2	Sharing experiences on responsiveness to diversity within the palliative care project
	We share lessons and successes in engaging patients with a non-western migration background in our project with third parties. We share our experiences with other researchers, project teams, networks, partners, etc.; We encourage these parties to increase their projects’ responsiveness to (the growing) diversity of the patient population within palliative care.

### Instrument testing

#### Delphi study

##### Design

To test the content of the pilot instrument we conducted a Delphi study. A Delphi study is a structured process that combines expert opinion into group consensus in a series of questionnaires, or ‘rounds’ [14]. Our Delphi study consisted of two rounds.

We translated the content of the pilot instrument into a questionnaire and asked a panel of experts to score the questionnaire items’ relevance and feasibility on a 5-point scale (1:not relevant/feasible; 5:entirely relevant/feasible). Feedback on the results from round one was provided to the experts in the subsequent round with the aim to increase consensus. We used an a priori set consensus rate of 75%, where at least 75% of the scores given by the experts should be 4 ≤ (relevance), or 3 ≤ (feasibility) in order to uphold the item in the instrument. We used a lower cut-off score for feasibility to avoid having to exclude items that scored high on relevance but low on feasibility. Relevance was the main factor because the measures in the self-assessment instrument are to represent a certain standard of diversity responsiveness of palliative care projects.

##### Sample

We selected a purposive sample of experts who could provide varying perspectives, i.e. experts from various research fields, representatives of migrant organizations, healthcare professionals and subsidy providers. There is no clear guidance on the number of experts in a Delphi panel, but most studies use panels of between 15 to 35 people [15]. We invited 50 experts to circumvent issues of non-response and ensure a number between 15 and 35. The study was carried out in Dutch, permitting participation by experts from the Netherlands and Belgium.

##### Data collection

The Delphi panel ran from March to July 2018. Questionnaires were sent via email. The questionnaire consisted of 33 items, subdivided into four sections. One section with three items on the groups included in the instrument’s focus and three sections with items on the measures from the pilot instrument according to the project stages: set-up; execution, and follow-up. Some of the 20 measures contained multiple steps. We separated these into several items to diminish ambiguity in the questionnaire which resulted in a total of 30 items. The complete list of items is included as an attachment (See Additional file 1). We instructed experts to score items’ relevance and feasibility and give remarks at the end of each section.

We analysed the data from round one and removed items on which consensus had been reached from the questionnaire. We asked the experts to re-evaluate the remaining items in round two. For each of these items, experts received their own score and the overall mean score. Experts were also asked to share any suggestions they had for alternative formulation of the items.

##### Data analysis

The Delphi method produced both quantitative and qualitative data. We exported the quantitative data to SPSS to calculate mean scores and consensus rates. We analysed the qualitative data using thematic analysis [16]. Authors KS, MT and JS reviewed the data to identify key themes and subthemes. These were used in order to adjust the questionnaire for round two as well as inform the overall analysis.

#### Usability testing

##### Design

To be able to test and refine the instrument we adopted methods of usability testing [17]. Seven projects from the national program for palliative care innovation used our instrument to conduct a self-assessment while we were present for observation. Once, the lead researcher completed the self-assessment individually and a concurrent think aloud protocol was used. In this method participants verbalize their thoughts while performing tasks to evaluate an artifact [17]. In the other cases the research team completed the assessment together and constructive interaction was used as a method to test usability. In this method participants work together in performing a task, thereby verbalizing their thoughts through interaction [17]. Following the self-assessment we held semi-structured interviews with the lead researchers to gather input on their experience and discuss the usability of the instrument in improving diversity responsiveness of palliative care projects.

##### Sample

We selected a convenience sample of projects from the national program for palliative care innovation through one of the palliative care consortia in the Netherlands. Nonetheless, the sampled projects were diverse in focus and status of implementation. The projects aimed to 1) develop, implement and evaluate an advance care planning intervention for primary care practice; 2) develop, implement and evaluate a seamless palliative care trajectory between different care settings; 3) evaluate and strengthen a procedure to improve palliative homecare where GPs, community nurses and palliative care consultants work together to identify and meet the needs of palliative patients; 4) assess the palliative care needs and barriers to care for homeless people; 5) study goals set by patients with metastatic lung cancer and their physicians before treatment with chemotherapy, targeted therapy or immunotherapy and whether these goals are met; and 6 & 7) two projects aimed to strengthen shared decision-making at the end of life by developing and implementing shared decision-making tools, as well as methods to engage all stakeholders in shared-decision making in palliative care. Five of these projects had been involved in the development of the pilot instrument. We purposively included two additional projects (No. 6 & 7) because they were yet to start and could offer distinct insights from that stage.

##### Data collection

MT observed the self-assessment and subsequently interviewed the projects’ lead researchers about the usability of the instrument. All self-assessments, the concurrent think-aloud and constructive interaction procedures, and interviews were recorded and transcribed verbatim.

##### Data analysis

We analysed the transcripts of self-assessment and interviews using thematic analysis [16]. We searched for components of usability, i.e. learnability (how well can users complete self-assessment when they use the instrument for the first time?), error rate (how many errors do users make, how severe are these errors?), satisfaction (how pleasant is it to use the instrument), and  applicability (how do users prefer to apply the self-assessment) [18]. We used the results from this analysis to further develop the self-assessment instrument. We additionally identified themes concerning the effect of the self-assessment on diversity responsiveness of palliative care projects.

## Results

### Delphi study

Twenty-two experts completed the questionnaire in round one (44% response rate). Expertise varied from the field of palliative care (6), diversity in healthcare (5), migrant health (3), culture sensitive care (2), patient participation (1), or other (3). Two experts reported overlapping areas of expertise (i.e. palliative care for migrant patients).

The complete list of questionnaire items with mean scores and percentages is included as an attachment (see Additional file 1.). In round one the experts reached the consensus rate of 75% on 26 out of 33 items, with regards to *relevance*. With regards to *feasibility*, the consensus rate of 75% was reached for all but one item (item 23: on diversity in the workforce). However, qualitative analysis indicated that experts did debate feasibility as it was dependent on factors such as availability of means or data.

*“In my opinion many of the abovementioned aspects [items] are relevant, but the feasibility is often concerned with time and money. I am not sure how costly each individual measure is, but I think making a case for the relevance (by thorough research and lobby) can increase feasibility.” (2018.12.1 Expert)*

Furthermore, experts agreed on the need for increased responsiveness to diversity and recommended to, in addition to migrants, include other groups at risk of being underrepresented. Some experts advised an intersectional approach. Intersectionality [19] can be used as an analytic framework to identify how social categorizations such as ethnicity, socioeconomic status or gender create overlapping and interdependent systems of discrimination or disadvantage that lead to exclusion. However, experts did mention intersectionality is a complex concept and a truly intersectional approach might be difficult to achieve within palliative care projects. Based on these results we constructed a new description of diversity responsiveness, extending our focus to migrants and other vulnerable groups, and asked the expert panel for consensus on this description in round two.

Sixteen experts completed round two. Expertise varied from the field of palliative care (4), diversity in healthcare (4), migrant health (3), culture sensitive care (2) or other (3). Experts were asked to re-evaluate seven items with regards to *relevance,* which was the main factor because the measures in the self-assessment instrument are to represent a certain standard of diversity responsiveness of palliative care projects. The panel reached the consensus rate of 75% for one additional item (item 22: on training in cultural competence).

One item was the new description of diversity responsiveness. Experts generally agreed with the new description and offered slight additions. E.g. instead of the term vulnerable groups it was suggested to use the term underserved groups. And while endorsing the extended focus, experts stressed that in deliberating the intersecting factors that put patients at risk of being underserved, the variety of factors at play for migrant patients necessitates continued special consideration of this group. This led to a final description of diversity responsiveness as: “Diversity responsiveness of palliative care projects entails deliberation of all intersecting factors that may cause patients to be underrepresented in palliative care research and underserved by palliative care services. ... These include factors such as educational background, socioeconomic status, physical or mental disability, age, sex, gender, sexual orientation, language, religion, culture, ethnicity and migration history. We ask special consideration of factors at play for migrant patients.” (see Additional file 2).

The remaining five items did not reach the cut-off score (items 4 & 5: on gaining insight into the diversity of the patient population under study and describing this in the project proposal; item 8: on registering patient ethnicity to monitor the use of the intervention under study; item 23: on encouraging diversity in the workforce of the healthcare organisations involved in the study; and, item 31: on adding tools to enhance culture sensitivity product from the project). These five items were removed from the final list of items for the self-assessment instrument. Comments by the Delphi experts indicated that these items were deemed not reasonably achievable or not desirable. In fact, mean scores for these items with regards to relevance were slightly lower than in round one. The final list of items was shared with the Delphi panel.

For the final self-assessment instrument we eventually merged two items and included one as a recommendation rather than a measurable element. The final instrument thus consists of 23 measures of diversity responsiveness.

### Usability testing

Results from usability testing gave insight into learnability, error-rate, satisfaction and applicability and the effect of the self-assessment on diversity responsiveness of palliative care projects. We used these to further develop the self-assessment instrument. The observed usability issues and subsequent adaptations to the instrument can be found in Table 2. We will discuss findings with regard to satisfaction, applicability and effect.

**Table 2:** Observed barriers and adaptations based on usability testing

	Barrier	Adaptations
Introduction		
^a^	Introduction text too lengthy and complicated.	We shortened the text, simplified the language and made the focus of the introduction more practical.
^a^	Discussion about the exemption of people from Indonesia in the definition of ‘non-western migrants’.	We stopped using the distinction non-western.
Items		
^a^	Difference between sentences in bold and normal writing unclear.	We either integrated the statements into one measure, or divided the statements into two measures.
^a^	Sentence *“based on ethnicity and intersecting factors”* Unclear what is meant by intersecting factors.	We take into account factors that contribute to underrepresentation of population groups, such as language, religion, culture, ethnicity migration history, education level, socioeconomic status, physical or intellectual disability, age, sex, gender and sexual preference.
^b^	*We include ethnicity as a variable in research in our project*. On one occasion it was mentioned that ethnicity was indeed registered, but not otherwise looked at.	This item was excluded as a result of the Delphi study.
^b^	*We monitor differences in care needs* *With the help of existing monitors and registrations used in care we will evaluate the care for migrant patients and their relatives/caretakers before, during and after the project.* This was not necessarily done with the help of *existing* monitors and registrations.	Rephrased to: To monitor the engagement of a diverse patient population We consider factors^a^ that contribute to underrepresentation of population groups in research within our project, with the aim to determine whether differing outcomes between groups depend on these factors. For example as a subgroup analysis. ^a^Language, religion, culture, ethnicity, migration history, educational level, socioeconomic status, physical or mental disability, age, sex, gender and sexual preference.
^b^	*We gather input from patients (panels or organizations) at differing project stages.* It was unclear that this included input from underrepresented groups.	Rephrased to: We gather input from patients, patient panels or patient organizations relevant to our project, in all stages of the project.
^b^	*We consider the patients we do and do not reach when selecting healthcare organizations where our study will be implemented. There is geographical variety …* Variety in locations was often aimed for, for other reasons.	Rephrased to: We implement the project in differing locations to guarantee access for underrepresented groups.
^b^	*Patient information materials about and within our project are understandable.* Too open for own interpretation.	Rephrased to: We test whether patient information materials used in our project are appropriate in terms of language, (health) literacy level, and culture sensitivity.
^b^	*With our project we contribute to improved access to palliative care for patients with a non-western migrant background.* Too open for own interpretation.	Rephrased to: We ensure that patient participation in our project improves access to palliative care for underrepresented / underserved patients and their communities, for instance through patient education, patient navigation or community outreach.
^b^	*We pay attention to responsiveness to patients with a non-western migrant background in our recommendations or implementation of the project on new locations.* Unclear whether this included implementation outside of the project.	Rephrased to: We account for underrepresented groups in the recommendations or roll-out of our project.
Score		
^a^	Difference between the five options (no, hardly, partially, mostly, completely) was hard to distinguish.	We changed to a three point score (no, partially, completely).
^a^	It was unclear what the options represented.	We changed to a three point score and included a description of scoring options above the tick boxes (no, partially, completely).
^a^	It was unclear that items could be not applicable, and could be scored as such.	We included the option ‘not applicable’ to the description of scoring options above the tick boxes and distinguishes it by using bold letters. (No, partially, completely, N.A.)
Tips		
^c^	Flipping pages between the measures and the tips.	Moved all the tips and recommendations to an attachment.
^a^	It was unclear which tips and recommendations dealt with which subject.	We moved all the tips and recommendations to an attachment and added subject headings
Summary		
^a^	The question ‘Where does the project stand?’ in terms of *responsiveness* was misinterpreted and answered in terms of *project stage.*	We removed the textbox and instead included a smaller textbox for actions at the end of each measure.
	Textbox where a summary of the evidence and measure for improvements could be written down was not used.	We removed the textbox and instead included a smaller textbox for actions at the end of each measure.
Lay-out		
^c^	Confusion about how far along the team was, if everyone was on the same page.	Added page numbers.
^c^	Landscape orientation was unpleasant.	We changed lay-out to portrait orientation.
Other		
	Questions about diversity amongst healthcare professionals arose.	We added tips and recommendations on this topic in the attachment.
^a^	Unfamiliar terms: intersectionality, culture-sensitive, self-organization.	Instead of, or when using the terms we describe what they stand for.
^c^	Discussion on whether we cover diversity with this instrument, which mostly (solely) targets non-westerns migrants.	We widened the scope of the instrument.

^a^ Learnability: how well can users complete self-assessment when they use the instrument for the first time?

^b^ Error rate: how many errors do users make, how severe are these errors?

^c^ Satisfaction: how pleasant is it to use the instrument?

#### Satisfaction

Overall, users were satisfied with the self-assessment. They did, however, report some concern about the amount of text and use of difficult or unfamiliar terms.

*“I had indeed already read it … and I have to say, it didn’t come easily. … That has to do with words such as intersectionality and there were some other terms. But otherwise it was quite accessible.”* (*2018.13 Researcher*)

Some users mentioned they found the assessment confronting, because it uncovered the paucity in measures they had taken to be responsive. However, the subsequent discussion that lead to actions being formulated was regarded as very positive.

*Yes, indeed, I found that to be very nice! … The actions are supported by the group. Especially because it is so confronting, in a certain way, that you see ‘we don’t do any of this’. And there was no one, not anyone that said ‘we don’t have to do it’ and is completely … I don’t think you will convince those. But in this group I really liked that it resulted in supported actions.” (2018.13 Researcher)*

#### Applicability

Whether diversity responsiveness of palliative care projects can be reasonably achieved with this instrument was said to depend on timing of the self-assessment and whether researchers remembered to use the instrument. Users indicated they prefer to conduct the self-assessment during the early stages of the project, to be able to incorporate actions into project plans. Users saw the potential of using the instrument in differing project stages, but were uncertain about the likelihood of actually repeating the self-assessment. This depended on whether they were reminded, or even obliged, to do so. For instance because the self-assessment is embedded in a process of acquiring funding.

*“I think that the need for this instrument especially exists when writing proposals. That people’s attention is drawn to the instrument when they are writing proposals. … I think it would be useful for people. Because, usually, you merely do something based on your own vision and what you know as a team. But usually there is a lot more, and this [the instrument] can help you with that.” (2018.10 Researcher)*

As the Delphi study and usability testing occurred simultaneously no adjustments had been made to the pilot instrument based on the results from the Delphi study. Interestingly, the instrument’s focus on migrant patients similarly elicited discussion. Users indicated that underserved groups in palliative care other than migrant patients should be included. For example, palliative care has for many years focused mainly on oncology patients as a result of which patient groups with other conditions have been underserved. Moreover, educational level, (health) literacy level and religion were mentioned as factors that needed consideration. It was suggested that broadening the focus would increase the applicability of the instrument.

*“You’re not going to do all of them. It’s as simple as that. … If you target this towards low literacy, I’m just picking something, and not specifically towards cultural diversity. Which is indeed very important, and can be an aspect of it, but if it is not only targeted towards that … I am not going to use one tool for patients with a non-western background, another for … The broader the applicability of a tool … ” (2018.12 Researcher)*

#### Effect

We found the self-assessment mainly functioned as a way to raise awareness about diversity amongst palliative care patients. The instrument offered a structured way to think about the diversity responsiveness of a project.

*“The good thing about this instrument, in your project proposal you do mention diversity, but this forces you to think more carefully about how you are going to incorporate it in your project. And it points out your shortfalls.” (2018.14 Researcher)*

During all self-assessments users identified actions they could take to improve diversity responsiveness in current or future projects. Users expressed an ambition to make these improvements, but did so carefully.

*Yes, well I would be interested to conduct an additional small-scale qualitative research … I really like the idea. But we’re not pinned down on what we promise here, are we? (2018.15 Researcher)*

## Discussion

With this study we aimed to develop and test a self-assessment instrument to help researchers assess and find ways to improve their palliative care projects’ responsiveness to diversity in light of the aging migrant population. By implementing all changes and omitting some items the self-assessment instrument includes a list of 23 measures to be taken at varying stages of a project and targets all groups at risk of being underrepresented in palliative care research and underserved by palliative care services. The measures cover topics important during project set-up: *describing the diversity of the patient population in the project proposal; monitoring engagement of a diverse patient population; establishing a representative project team;* project execution: *patient and caregiver participation; access to and quality of care within the project* and, follow-up: *responsive implementation of project results; and, sharing experiences on responsiveness in palliative care projects.* The complete final instrument is included as an attachment (see Additional file 2).

Our findings indicate that underserved groups in palliative care other than migrant patients should be included in the instrument’s focus. This was stressed by both the experts involved in the Delphi study and the researchers engaged in usability testing. Indeed, a shift away from single target groups has been the trend in thinking about responsiveness [20, 21]. Our final instrument is now more in accordance with the approach of the equity standards, which targets all groups at risk of inequities in health and healthcare in response to the increasing differentiation of diversity [22] and the need to recognize multiple-diversity needs, as individual needs are generally expressed by the intersection of many factors such as origin, class, gender, age and ability [23].

While our Delphi results indicate a need to increase awareness of the way in which intersections of differences put certain groups at risk of being underrepresented in palliative care research and practice, usability testing showed researchers were unfamiliar with the concept of intersectionality and were more practically motivated to extend the focus beyond migrant patients. Extending the focus would increase the instrument’s applicability and projects that worked to improve palliative care for underserved groups other than migrants liked to see their efforts reflected in the self-assessment. Indeed, intersectionality is often seen as difficult to implement in, especially quantitative, health research methodology [24]. Our final instrument, therefore, asks for consideration of the varying dimensions of diversity but leaves the choice for an intersectional approach to the researchers. Furthermore, in the deliberation of all factors that may cause patients to be underrepresented in palliative care research we do continue to ask for special consideration of factors at play for migrant patients.

Results from usability testing show researchers were able to conduct the self-assessment and were satisfied with the process. The self-assessment helps raise awareness and offers a structured way to think about the diversity responsiveness of a project. Researchers should be attentive to the risk of using the self-assessment to pick and choose actions, rather than using it to make structural improvements in diversity responsiveness. Embedding the self-assessment in the process of acquiring funding can encourage future use. Research has shown that researchers whose funding included a mandate on minority participant inclusion adopted more, and more active recruitment strategies in recruiting minorities [25]. At the same time, increased engagement of underrepresented populations has been found to be beneficial to secure funding, design study protocols, increase enrolment rates and select relevant outcomes, resulting in reporting that is more meaningful and understandable for patients and the community [26].

To our knowledge, we are the first to apply the content of the equity standards, an instrument for the context of healthcare organisations, to the context of health related research. In an elaborate development phase we adjusted the content of the equity standards to fit the context of research, specifically research in palliative care. Our findings suggest the equity standards are applicable in this context and form a good basis for a self-assessment instrument to be used to improve diversity responsiveness of palliative care research. As such our self-assessment instrument may complement the MORECare statement, a checklist issued to set a standard for research in end of life care [27]. The MORECare statement does include statements on engaging patients but puts more emphasis on the vulnerable position of patients approaching the end of life, with statements as “Collaborate with patients and caregivers in the design of the study, vocabulary used in explaining the study, consent procedures and any ethical aspects.” or “Ensure proportionality in patient and caregiver information sheets, appropriate to the study design and level of risk, as excessive information in itself can be quite tiring/distressing for very ill individuals.”. Our self-assessment instrument may additionally provide a set of standards to help engage diverse patients.

Our findings are limited by the fact that our self-assessment instrument was developed in the context of the Netherlands and the national program for palliative care innovation (*Palliantie. Meer dan Zorg*). The particular needs of migrants and other underserved patients will similarly need to be considered in palliative care research in other European countries, and further research is needed to be able to generalize our findings and make the self-assessment instrument applicable to other contexts. Our findings are additionally limited by the fact that four of the projects involved in usability testing performed at least part of the self-assessment in retrospect because their projects were in a well-advanced project stage. This may have influenced users’ readiness to identify areas for improvement. Furthermore, the use of think aloud methods during usability testing required MT to attend the self-assessment. Her presence may have influenced the research teams’ efforts and prompted socially desirable answers. A next step may be to conduct an implementation study to monitor the actions taken by researchers involved in the study and evaluate their effect on the engagement of underrepresented populations.

## Conclusion

We have developed and tested a self-assessment instrument which can be used by researchers involved in palliative care projects to assess and find ways to improve their projects’ diversity responsiveness. It helps raise awareness and offers a structured way to think about diversity responsiveness and identify areas for improvement. We recommend performing the self-assessment during the early stages of a project, with the option to repeat it in each progressing stage. Improving responsiveness of projects will help researchers tackle the problem of underrepresentation of migrants and other underserved populations and enable them to address questions on differing needs and appropriateness of services in palliative care as part of their research.

## Supplementary information

**Additional file 1.** Final list of Delphi questionnaire items with mean scores and percentages. The Delphi questionnaire was established by translating the content of a pilot self-assessment instrument for diversity responsiveness in palliative care projects into a questionnaire. The questionnaire consisted of 33 items, subdivided into four sections. One section with three items on the groups included in the instrument’s focus and three sections with items on the measures from the pilot instrument according to the project stages: set-up; execution, and follow-up. The questionnaire was sent to a panel of experts who were asked to score the questionnaire items’ relevance and feasibility on a 5-point scale (1:not relevant/feasible; 5:entirely relevant/feasible). A priori set consensus rate of 75% were used, where at least 75% of the scores given by the experts should be 4 ≤ (relevance), or 3 ≤ (feasibility) in order to uphold the item in the instrument. Listed here are the final items, after revisions suggested by experts, the mean scores and consensus rate percentages.

**Additional file 2. **Self-assessment instrument Diversity responsiveness in palliative care projects. The aim of this self-assessment instrument is to help researchers establish a project responsive to the diversity of the palliative care patient population. It helps to assess your project’s responsiveness at present and identify areas for improvement. The instrument is structured according to three project stages: 1) the project set-up; 2) the project execution; 3) project follow-up. By means of 23 diversity responsiveness measures you assess your project’s responsiveness at present. Every measure has three response options indicating the level of implementation in the project (no, partially, completely). You can specify actions for improvement on each measure. This self-assessment instrument has been developed as part of the project “*Palliatieve zorgprojecten langs de diversiteitsmeetlat”*, an implementation project of the program *Palliantie. Meer dan Zorg* funded by The Netherlands Organization for Health Research and Development. The development of self-assessment instrument is based on the *standards for equity in healthcare for migrants and other vulnerable groups* [13] and additional of literature research, expert consultation and a usability study.

## Data Availability

The datasets generated and analysed during this study are available from the corresponding author on reasonable request.
