# Supplementary material for: How to help researchers in palliative care improve responsiveness to migrants and other underrepresented populations: developing and testing a self-assessment instrument
Source: BMC Palliat Care. 2019 Oct 21;18:83. doi: 10.1186/s12904-019-0470-1 (PMC6805674; doi:10.1186/s12904-019-0470-1)
Supplement: Supplementary file 1 — Additional file 1. Final list of Delphi questionnaire items with mean scores and percentages. The Delphi questionnaire was established by translating the content of a pilot self-assessment instrument for diversity responsiveness in palliative care projects into a questionnaire. The questionnaire consisted of 33 items, subdivided into four sections. One section with three items on the groups included in the instrument’s focus and three sections with items on the measures from the pilot instrument according to the project stages: set-up; execution, and follow-up. The questionnaire was sent to a panel of experts who were asked to score the questionnaire items’ relevance and feasibility on a 5-point scale (1:not relevant/feasible; 5:entirely relevant/feasible). A priori set consensus rate of 75% were used, where at least 75% of the scores given by the experts should be 4 ≤ (relevance), or 3 ≤ (feasibility) in order to uphold the item in the instrument. Listed here are the final items, after revisions suggested by experts, the mean scores and consensus rate percentages. [file 12904_2019_470_MOESM1_ESM.docx]

Additional file 1. Final list of Delphi questionnaire items with mean scores and percentages.

|  |  | **Relevance** | | | | **Feasibility** |
| --- | --- | --- | --- | --- | --- | --- |
|  |  | **Round 1**  Mean  (SD) | **Round 1**  Consen-sus rate % | **Round 2**  Mean  (SD) | **Round 2**  Consen-sus rate % | **Round 1**  Mean  (SD) |
|  | **Section 1** |  |  |  |  |  |
| 1 | *Responsiveness to diversity entails deliberation of factors such as age, language, socioeconomic status, disability, sex, gender, culture and ethnicity, as well as the interacting effect of these factors which may contribute to social exclusion. ** | 4.7 (0.5) | 100 |  |  | 3.5 (1.0) |
| 2 | *To overcome inequity in access to palliative care we must, in addition to considering specific underrepresented groups, pay attention to the intersection of all factors which may contribute to social exclusion.** | 4.5 (0.7) | 91 |  |  | 3.2 (1.1) |
| 3 | *In palliative care projects we should first address underrepresentation of migrants and ethnic minorities and address inclusion of other vulnerable groups at risk of social exclusion secondly.** | 2.9 (1.1) | 33 |  |  | 3.2 (1.0) |
|  | **Section 2. Project set-up** |  |  |  |  |  |
| 4 | *To gain insight into the demographic composition of the patient population based on abovementioned factors, either via literature or empiric research.+* | 3.8 (0.9) | 57 | 3.7 (0.7) | 69 | 4.1 (0.9) |
| 5 | *To (explicitly) describe the diversity of the patient population in a project proposal or project plan.+* | 3.9 (1.1) | 73 | 3.8 (0.9) | 63 | 3.7 (1.2) |
| 6 | *To justify choices (not) to make the project responsive to diversity in the project proposal, these include choices with regards to budget, etc.* | 4.2 (0.9) | 77 |  |  | 4.0 (1.1) |
| 7 | *To describe the implications for the outcome of the project when the choice is made not to make the project responsive to diversity, in the project proposal.* | 4.2 (0.7) | 82 |  |  | 3.8 (0.9) |
| 8 | *To register patient ethnicity before, during and after the palliative care project with the aim to monitor the appropriateness of the care innovation for migrant patients and their informal caretakers. +* | 3.6 (1.0) | 65 | 3.6 (1.0) | 69 | 3.0 (1.1) |
| 9 | *To consider factors that contribute to underrepresentation of population groups in research within the project, with the aim to determine whether differing outcomes between groups depend on these factors. ×* | 4.3 (0.9) | 82 |  |  | 3.5 (1.0) |
| 10 | *To plan subgroup analyses in the research plan with the aim to , with the aim to determine whether differing outcomes between groups depend on factors that contribute to underrepresentation of population groups. ×* | 4.1 (0.7) | 77 |  |  | 3.1 (1.1) |
| 11 | *To engage partners (experts, patient representatives, etc.) whom are knowledgeable on the topic of palliative care for underserved groups in our project team to secure this knowledge within out project.* | 4.3 (0.9) | 81 |  |  | 4.0 (1.0) |
| 12 | *To work together with partners (experts, patient representatives, etc.) whom are knowledgeable on the topic of palliative care for underserved groups to secure this knowledge within out project.* | 4.3 (0.8) | 80 |  |  | 3.7 (1.1) |
|  | **Section 3. Project execution** |  |  |  |  |  |
| 13 | *To gather input from patients, patient panels, or patient organisations relevant to our project in all stages of the project.* | 4.3 (0.7) | 86 |  |  | 3.2 (0.9) |
| 14 | *To identify and overcome (experienced) barriers for patient participation by underrepresented groups.* | 4.6 (0.6) | 95 |  |  | 3.5 (0.8) |
| 15 | *To ensure that patient participation in our project improves access to palliative care for underrepresented / underserved patients and their communities, for instance through patient education, patient navigation or community outreach.* | 4.5 (0.9) | 86 |  |  | 3.8 (0.8) |
| 16 | *To implement the project in differing locations to guarantee access for underrepresented groups.* | 4.1 (0.8) | 76 |  |  | 3.7 (0.9) |
| 17 | *To stop using Dutch language proficiency as an inclusion criteria.°* | 4.4 (0.7) | 86 |  |  | 3.1 (0.8) |
| 18 | *To take into consideration (health) literacy levels, language ability, and culture when asking for informed consent.* | 4.5 (0.8) | 90 |  |  | 4.1 (0.8) |
| 19 | *To identify and overcome gatekeeper bias amongst parties responsible for inclusion of underrepresented patients within our project, for instance healthcare professionals.* | 4.5 (0.8) | 85 |  |  | 3.5 (0.6) |
| 20 | *To raise awareness amongst healthcare organizations and healthcare professionals involved in the project on the increasing diversity and amongst palliative care patients and their differing needs.¨* | 4.5 (0.8) | 90 |  |  | 4.3 (0.8) |
| 21 | *To actively inquire about barriers and concerns with regards to palliative care for underserved groups amongst healthcare organisations and healthcare professionals engaged in the project.¨* | 4.4 (0.8) | 90 |  |  | 4.2 (0.8) |
| 22 | *To offer healthcare organizations and healthcare professionals engaged in the project training in diversity responsive care practice, i.e. person-centered care which considers group factors that put individuals at risk of inequities in health and healthcare.* | 4.2 (1.2) | 70 | 4.3 (0.9) | 87 | 4.0 (1.0) |
| 23 | *To encourage healthcare organizations involved in the project to create diversity in the workforce, resembling the diversity of their patient population. +* | 4.0 (1.0) | 71 | 3.8 (0.8) | 56 | 2.9 (0.9) |
| 24 | *To test whether patient information materials used in our project are appropriate in terms of language, (health) literacy level, and culture sensitivity.* | 4.7 (0.6) | 95 |  |  | 4.0 (0.9) |
| 25 | *To test validity of our measurement instruments in terms of language, (health) literacy level and culture sensitivity.* | 4.2 (0.9) | 76 |  |  | 3.2 (0.8) |
| 26 | *To offer the possibility to complete consent procedures, questionnaires, or interviews orally and if necessary in the language of the patient.* | 4.5 (0.6) | 95 |  |  | 3.5 (1.0) |
|  | **Section 4. Project follow-up** |  |  |  |  |  |
| 27 | *To consult patient panels or patient organisations representative of the patient population in our project for interpretation of results from our project.* | 4.5 (1.0) | 90 |  |  | 4.0 (0.8) |
| 28 | *To share relevant findings concerning underrepresented groups as results of our project in a respectful manner.* | 4.7 (0.6) | 95 |  |  | 4.2 (0.8) |
| 29 | *To share findings concerning underrepresented groups with these groups in an understandable, appropriate manner.* | 4.6 (0.6) | 95 |  |  | 4.0 (0.8) |
| 30 | *To ensure responsiveness to diversity in the recommendations or rollout of our project.* | 4.6 (0.7) | 91 |  |  | 3.8 (1.0) |
| 31 | *To add tools which can help healthcare professionals enhance culture sensitivity to product/results from the project.+* | 3.9 (0.9) | 67 | 3.8 (0.8) | 69 | 3.6 (0.9) |
| 32 | *To share lessons and successes in ensuring responsiveness to diversity in our project with third parties (e.g. other researchers, project teams, networks, partners, etc.) to increase engagement of underrepresented groups in palliative care projects.* | 4.0 (0.6) | 95 |  |  | 4.2 (0.8) |
| 33 | *To raise* *awareness on the growing diversity of the patient population within palliative care amongst third parties (e.g. other researchers, project teams, networks, partners, etc.).* | 4.6 (0.6) | 95 |  |  | 4.2 (0.8) |

Formulation of all was items adjusted based on expert feedback (e.g. use of the term underrepresented groups instead of migrants and other vulnerable groups).

*Items 1-3 were combined to the final description of diversity responsiveness as: “Diversity responsiveness of palliative care projects entails deliberation of all intersecting factors that may cause patients to be underrepresented in palliative care research and underserved by palliative care services … These include factors such as educational background, socioeconomic status, physical or mental disability, age, sex, gender, sexual orientation, language, religion, culture, ethnicity and migration history. We ask special consideration of factors at play for migrant patients.”

+ Items were removed from the list of items that formed the basis for the final self-assessment instrument.

× Items were combined in the final self-assessment instrument to avoid overlap.

¨ Items were combined in the final self-assessment instrument to avoid overlap.

*°* This item was included as a recommendation rather than a measurable item.
