# Supplementary material for: How to help researchers in palliative care improve responsiveness to migrants and other underrepresented populations: developing and testing a self-assessment instrument
Source: BMC Palliat Care. 2019 Oct 21;18:83. doi: 10.1186/s12904-019-0470-1 (PMC6805674; doi:10.1186/s12904-019-0470-1)
Supplement: Supplementary file 2 — Additional file 2. Self-assessment instrument Diversity responsiveness in palliative care projects. The aim of this self-assessment instrument is to help researchers establish a project responsive to the diversity of the palliative care patient population. It helps to assess your project’s responsiveness at present and identify areas for improvement. The instrument is structured according to three project stages: 1) the project set-up; 2) the project execution; 3) project follow-up. By means of 23 diversity responsiveness measures you assess your project’s responsiveness at present. Every measure has three response options indicating the level of implementation in the project (no, partially, completely). You can specify actions for improvement on each measure. This self-assessment instrument has been developed as part of the project “Palliatieve zorgprojecten langs de diversiteitsmeetlat”, an implementation project of the program Palliantie. Meer dan Zorg funded by The Netherlands Organization for Health Research and Development. The development of self-assessment instrument is based on the standards for equity in healthcare for migrants and other vulnerable groups [13] and additional of literature research, expert consultation and a usability study. [file 12904_2019_470_MOESM2_ESM.docx]

**Self – assessment instrument**

Diversity responsiveness in palliative care projects

## *Introduction*

Research and innovation that strives for palliative care to better fit the needs and wishes of patients and their families facing the problems associated with the end of life, requires consideration of the question whom will benefit from improved palliative care. By becoming responsive to the diversity of the patient population the outcomes of palliative care research and innovation will be beneficial to *all* service users.

The aim of this self-assessment instrument is to help you establish a project responsive to the diversity of the palliative care patient population. This self-assessment instrument helps you to assess your project’s responsiveness at present and identify areas for improvement.

Responsiveness to diversity entails deliberation of all intersecting factors that may cause patients to be underrepresented in palliative care research and underserved by palliative care services. i.e. those patients for whom access to and quality of care are inadequate. These include factors such as educational background, socioeconomic status, physical or mental disability, age, sex, gender, sexual orientation, language, religion, culture, ethnicity and migration history. You consider the factors relevant to the patient population of your project, and in addition we ask special consideration of factors at play for migrant patients.

## *Instructions*

This self-assessment instrument is structured according to three project stages: 1) the project set-up; 2) the project execution; 3) project follow-up. By means of twenty-three diversity responsiveness measures you assess your project’s responsiveness at present. Every measure has three response options indicating the level of implementation in the project (no, partially, completely). You can specify actions for improvement on each measure. We recommend conducting the self-assessment with the project team. Self-assessment will take between twenty and thirty minutes. Attached to the self-assessment instrument you will find a list of recommendations that will help you increase your project’s responsiveness to diversity.

## *Accountability*

This self-assessment instrument has been developed as part of the project *palliatieve zorgprojecten langs de diversiteitsmeetlat*, an implementation project of the program *Palliantie. Meer dan Zorg* funded by The Netherlands Organization for Health Research and Development. The development of self-assessment instrument is based on the *standards for equity in healthcare for migrants and other vulnerable groups* (HPH Taskforce MFH, 2014) and additional of literature research, expert consultation and a usability study.

**Colofon**

**Self-assessment instrument |** Diversity in palliative care projects

Amsterdam, 2018

This self-assessment instrument has been developed as part of the project *palliatieve zorgprojecten langs de diversiteitsmeetlat*, an implementation project of the program *Palliantie. Meer dan Zorg* funded by The Netherlands Organization for Health Research and Development.


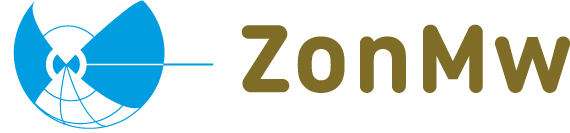


Amsterdam UMC, University of Amsterdam

Dept. of Public Health, Amsterdam Public Health Research Institute

Meibergdreef 9, 1100 DD Amsterdam, The Netherlands

Amsterdam UMC, Vrije Universiteit Amsterdam

Dept. of Public and Occupational Health, Amsterdam Public Health Research Institute

De Boelelaan 1117, 1081 BT Amsterdam, The Netherlands.

1. The project set-up

The first step is to gather insight into the diversity of the project’s patient population. Consider during the set-up of the project if, and if so, which, extra efforts are needed to make the project and the project outcome responsive to diversity. Plan during the set-up of the project furthermore how to monitor the appropriateness of the palliative care innovation for all patients.

**Goal: To arrive at a project set-up which contributes to equitable palliative care innovation**

| **1.1** | **Describe the diversity of the patient population in the project proposal**  **No, partially, completely**, N.A. | | **Actions** |
| --- | --- | --- | --- |
| We determined which groups are at risk of being underrepresented in our project and need consideration. | | □ □ □ □ |  |
| We justify our choices (not) to make the project responsive to diversity in our project proposal, these include choices with regards to budget, etc. | | □ □ □ □ |  |
| We describe the implications for the outcome of the project when we choose not to make the project responsive to diversity, in our project proposal. | | □ □ □ □ |  |

| **1.2** | **Monitoring the engagement of a diverse patient population**  **No, partially, completely**, N.A | | **Actions** |
| --- | --- | --- | --- |
| We consider factors* that contribute to underrepresentation of population groups in research within our project, with the aim to determine whether differing outcomes between groups depend on these factors. For example as a subgroup analysis.  *Language, religion, culture, ethnicity, migration history, educational level, socioeconomic status, physical or mental disability, illness, age, sex, gender and sexual preference. | | □ □ □ □ |  |

| **1.3** | **Establishing a representative project team**  **No, partially, completely**, N.A | | **Actions** |
| --- | --- | --- | --- |
| We work together with partners (experts, patient representatives, etc.) whom are knowledgeable on the topic of palliative care for underserved groups to secure this knowledge within out project. | | □ □ □ □ |  |
| We engage partners (experts, patient representatives, etc.) whom are knowledgeable on the topic of palliative care for underserved groups in our project team to secure this knowledge within out project. | | □ □ □ □ |  |

2. The project execution

There is continuous effort to ensure responsiveness to diversity during the project. Barriers in access to the palliative care project and palliative care services more generally are identified and overcome. Patients and their families are actively engaged to help achieve this.

**Goal: To execute the project in such a way that all patients have equal opportunities to participate in the project and receive quality care during and after the project.**

| **2.1** | **Patient and family participation**  **No, partially, completely**, N.A | | **Actions** |
| --- | --- | --- | --- |
| We gather input from patients, patient panels, or patient organisations relevant to our project in all stages of the project. | | □ □ □ □ |  |
| We identify and overcome (experienced) barriers for patient participation by underrepresented groups. | | □ □ □ □ |  |
| We ensure that patient participation in our project improves access to palliative care for underrepresented / underserved patients and their communities, for instance through patient education, patient navigation or community outreach. | | □ □ □ □ |  |

| **2.2** | **Access to (care within) the project**  **No, partially, completely**, N.A | | **Actions** |
| --- | --- | --- | --- |
| We implement the project in differing locations to guarantee access for underrepresented groups. | | □ □ □ □ |  |
| We identify and overcome gatekeeper bias amongst parties responsible for inclusion of underrepresented patients within our project, for instance healthcare professionals. | | □ □ □ □ |  |
| We take into consideration (health) literacy levels, language ability, and culture when asking for informed consent. | | □ □ □ □ |  |
| We test whether patient information materials used in our project are appropriate in terms of language, (health) literacy level, and culture sensitivity. | | □ □ □ □ |  |
| We test validity of our measurement instruments in terms of language, (health) literacy level and culture sensitivity. | | □ □ □ □ |  |
| We offer the possibility to complete consent procedures, questionnaires, or interviews orally and if necessary in the language of the patient. | | □ □ □ □ |  |

| **2.3** | **Quality of care within the project**  **No, partially, completely**, N.A | | **Actions** |
| --- | --- | --- | --- |
| We actively inquire about barriers and concerns with regards to palliative care for underserved groups amongst healthcare organisations and healthcare professionals engaged in the project. | | □ □ □ □ |  |
| We offer healthcare organisations and healthcare professionals engaged in the project training in *diversity responsive* care practice.  * *Diversity responsive* care is person-centred care which considers group factors that put individuals at risk of inequities in health and healthcare. | | □ □ □ □ |  |

3. The project follow-up

After the project has ended the improved palliative care, including extra efforts to ensure it is responsive to diversity, is sustained. The experience of working towards responsive palliative care innovation are shared with other project teams and researchers.

**Goal: To stimulate efforts to ensure responsiveness to diversity in palliative care both after and beyond the project.**

| **3.1** | **Implementation of project results**  **No, partially, completely**, N.A | | **Actions** |
| --- | --- | --- | --- |
| We consult patient panels or patient organisations representative of the patient population in our project for interpretation of results from our project. | | □ □ □ □ |  |
| We share relevant findings concerning underrepresented groups as results of our project in a respectful manner. | | □ □ □ □ |  |
| We share findings concerning underrepresented groups *with* these groups in an understandable, appropriate manner. | | □ □ □ □ |  |
| We ensure responsiveness to diversity in the recommendations or rollout of our project. | | □ □ □ □ |  |

| **3.2** | **Sharing experiences on responsiveness in palliative care projects**  **No, partially, completely**, N.A | | **Actions** |
| --- | --- | --- | --- |
| We share lessons and successes in ensuring responsiveness to diversity in our project with third parties (e.g. other researchers, project teams, networks, partners, etc.) to increase engagement of underrepresented groups in palliative care projects. | | □ □ □ □ |  |
| We work to raise awareness on the growing diversity of the patient population within palliative care amongst third parties (e.g. other researchers, project teams, networks, partners, etc.). | | □ □ □ □ |  |
